# Alternate Pembrolizumab Dosing Interval in Advanced NSCLC with PD-L1 TPS ≥ 50%: 3 Weekly Compared to 6 Weekly Dosing

**DOI:** 10.3390/curroncol29110685

**Published:** 2022-11-15

**Authors:** Lauren Jones, Rebekah Rittberg, Bonnie Leung, Aria Shokoohi, Alexandra Pender, Selina Wong, Zamzam Al-Hashami, Ying Wang, Cheryl Ho

**Affiliations:** 1 Department of Medical Oncology, BC Cancer, Vancouver, BC V5Z 4E6, Canada; 2 Faculty of Medicine and Dentistry, University of Alberta, Edmonton, AB T6G 2R7, Canada; 3 Department of Medical Oncology, The Royal Free NHS Foundation Trust, London NW3 2QG, UK; 4 Department of Medical Oncology, BC Cancer, Victoria, BC V8R 6V5, Canada; 5 Department of Medical Oncology, Sultan Qaboos Comprehensive Cancer Care and Research Center, Muscat 111, Oman

**Keywords:** PDL1, pembrolizumab, dosing schedule, NSCLC

## Abstract

Background: A fixed dose of 200 mg of pembrolizumab every 3 weeks (Q3W) is the standard of care for patients with stage IV non-small cell lung cancer (NSCLC) and PDL1 ≥50%. In April 2020, based on pharmacokinetic modeling without formal comparative studies, the FDA approved 400 mg every 6 weeks (Q6W). Pharmacokinetic studies also suggested comparable target engagement with weight-based and flat dosing for the respective schedules. The objective of this study was to determine if overall survival (OS) differs based on the Q3W vs. Q6W dosing schedule of pembrolizumab. Methods: BC Cancer patients with stage IV NSCLC and PDL1 ≥50% treated with pembrolizumab were retrospectively reviewed. Patients were treated with weight-based dosing, per institution standard, of pembrolizumab 2 mg/kg Q3W or 4 mg/kg Q6W. Patient demographics, treatment and outcome were recorded. Patients were assigned to Q3W or Q6W according to the schedule that was used for the majority of treatment (greater than 50%). Results: 718 patients with NSCLC and PDL1 ≥50% received first-line pembrolizumab between 2017 and2021, Q3W/Q6W dosing 677/41 patients. Baseline characteristics with respect to age, sex, smoking status, histology and performance status (PS) were similar between groups. In the multivariate model, including age, sex, PS and dosing schedule, the hazard ratio for death (HR) for OS Q3W vs. Q6W was 0.759 (*p* = 0.230). A 2:1 case-matched analysis for OS was performed, controlling for sex, age ± 5 years, PS and duration on pembrolizumab ± 2 months for Q3W vs. Q6W (*n* = 113) with a HR 0.834 (*p* = 0.500). Conclusions: There was no OS difference demonstrated with pembrolizumab dosing Q3W compared to Q6W in a multivariate analysis that included age, sex and PS. A case-matched analysis that controlled for these variables and for duration of treatment confirmed these findings. This study supports the use of Q6W pembrolizumab dosing, allowing for less frequent interactions with the medical system.

## 1. Background

Lung cancer is the most commonly diagnosed cancer in Canada, with projections of 233,900 new cases in 2022. Lung cancer also carries the highest fatality rate among cancer diagnoses, estimated to account for 25% of cancer-related deaths in Canada in 2022 due to the high rate of diagnosis in advanced stages [1]. The majority of lung cancer cases are non-small cell lung cancer (NSCLC). Fortunately, the treatment of stage IV NSCLC has evolved significantly, with multiple categories of systemic therapy, including chemotherapy, targeted therapy and immunotherapy [2,3]. Treatment selection is based on next-generation sequencing biomarker panel assays and the use of immunohistochemistry for program death ligand 1 (PDL1) expression.

The advancement of immunotherapy in the treatment of NSCLC has been due to the development of anti-PD1/PDL1 antibodies that facilitate tumor cell recognition by the immune system. The disruption of PD1 and PDL1 interaction allows for recognition of tumor cells by T-cells to mount an antitumor response, leading to apoptosis [4]. Use of these antibodies has been guided by tumor and/or immune cell PDL1 biomarker analysis, with the most common method relying on Tumor Proportion Score (TPS), the percentage of viable tumor cells showing partial or complete membrane staining at any intensity [5,6].

The standard of care treatment for patients with stage IV NSCLC and PD-L1 TPS ≥50% has evolved from platinum-based chemotherapy to pembrolizumab monotherapy 200 mg every 3 weeks (Q3W) due to the results found in KEYNOTE-024 [7]. The updated KEYNOTE-024 outcomes after 5 years of follow up demonstrated a median overall survival (OS) of 26.3 months in patients treated with pembrolizumab and 13.4 months with chemotherapy, a durable and clinically meaningful improvement. These data were also supported by the outcomes in the PDL1 TPS ≥50% subgroup in KEYNOTE-042 and as a drug class effect, as seen with cemiplimab in EMPOWER LUNG1 and IMpower110 [8,9,10].

Pharmacokinetic studies have suggested that weight-based and fixed dosing for Q3W and every-6-weeks (Q6W) dosing schedules of pembrolizumab have comparable target engagement [11]. In April 2020, the Food and Drug Administration (FDA) approved 400 mg Q6W based on pharmacokinetic modeling without formal comparative studies [12]. Health Canada and the European Medicines Agency have also approved Q6W dosing intervals of pembrolizumab in patients with cancer [13,14]. However, there is limited evidence to confirm that patients with advanced NSCLC achieve similar OS benefits with the longer interval dosing.

This study aimed to evaluate the OS based on the Q3W vs. Q6W pembrolizumab dosing schedule in real-world patients with NSCLC to confirm the expectations of pharmacokinetic modeling.

## 2. Methods

A retrospective study of patients with stage IV NSCLC and PDL1 ≥50% who were treated with first-line pembrolizumab monotherapy at BC Cancer in British Columbia (BC), Canada between 2017 and2021 was conducted. BC Cancer is a publicly funded program serving urban and rural patients across the province. The provincial program has oversight of cancer care for all residents of BC and thereby has complete oncology drug and radiotherapy records for all patients receiving cancer treatments.

Provincial electronic medical records and the Outcomes and Surveillance Integrated System (OaSIS) database were used to collect baseline characteristics. Systemic therapy details, including dosing interval and treatment duration, were collected retrospectively through the BC pharmacy database. All patients who received at least one dose of pembrolizumab were included.

All patients were treated with weight-based dosing of pembrolizumab 2 mg/kg Q3W or 4 mg/kg Q6W. Q6W dosing was approved at BC Cancer in April 2021. The dosing interval prescribed for patients was at the discretion of the treating medical oncologist. Assignment to Q3W or Q6W cohorts was based on the schedule used for the majority (greater than 50%) of the patient’s treatment.

The Chi-squared test, Fisher’s exact test and Kruskal–Wallis test were used for statistical analysis. OS was defined as the Stage IV NSCLC date of treatment initiation to date of death. OS was calculated using the Kaplan–Meier method, and the log-rank test was used for comparison. Cox-regression analysis was used for multivariate analysis. A case–control matching was conducted with an exact match for sex (male or female). Tolerance for age was ±5 years, there was an exact match for PS (0–1 and 2 or greater) and tolerance for duration of pembrolizumab therapy was ±2 months for Q3W matched to Q6W. Sampling without replacement was performed with the matching to be conducted to maximize execution performance. Statistical significance was defined as *p*-value < 0.05. IBM SPSS Statistics software, version 26 (IBM Corp, Armonk, NY, USA), was used for statistical analysis.

The ethics were approved by University of British Columbia-BC Cancer Research Ethics Board; H18-037444. Approval for a waiver of consent to extract and analyze the archival data from the database was granted.

## 3. Results

Between 2017 and 2021, 718 patients with NSCLC received first-line pembrolizumab: 677 patients who received Q3W dosing and 41 patients who received Q6W dosing. Baseline characteristics of the entire cohort include 54% female, 91% ever-smokers and 59% with PS 0–1. No statistical difference was detected in age, sex, histology or smoking status between the Q3W and Q6W groups (Table 1). The median durations of therapy were 4.2 months for Q3W (interquartile range (IQR) 1.4–12.6) and 6.2 months for Q6W (IQR 1.7–20.6). The median durations of follow up were 10.9 months for Q3W (IQR 3.7–23.4) and 14.5 months for Q6W (IQR 5.8–23.8).

**Figure 1:**
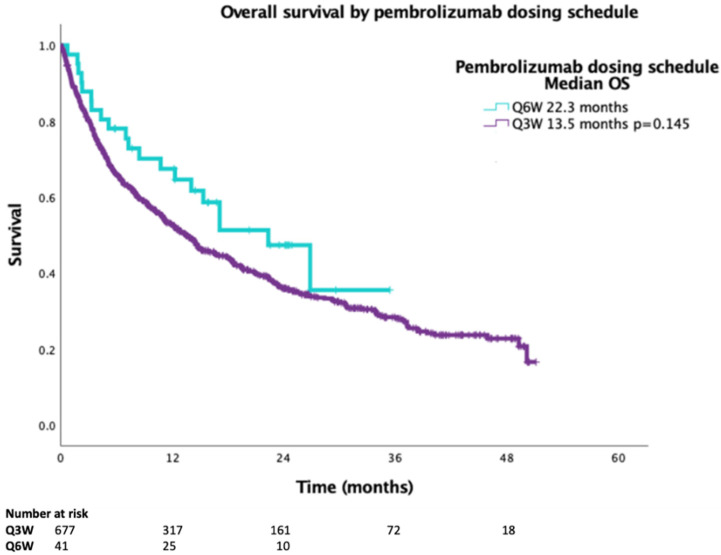
Overall survival for advanced NSCLC PDL1 TPS ≥ 50% patients treated with first-line pembrolizumab by treatment schedule (*n* = 718).

Median OS was 13.5 months in the Q3W pembrolizumab cohort (95% CI 11.370–15.702) and 22.3 months in the Q6W cohort (95% CI 13.799–30.817, *p* = 0.145), and the mean survival times were 21.0 months (95% CI 19.4–22.6) for Q3W and 20.6 months (95% CI 16.1–25.1) for Q6W. (Figure 1). In the multivariate model, including age, sex, PS and dosing schedule, poor PS was significant, with a hazard ratio (HR) of 2.1 for increased risk for death (*p* < 0.001). The HR for death for Q3W vs.Q6W was 0.718 (*p* = 0.147) (Table 2).

**Figure 2:**
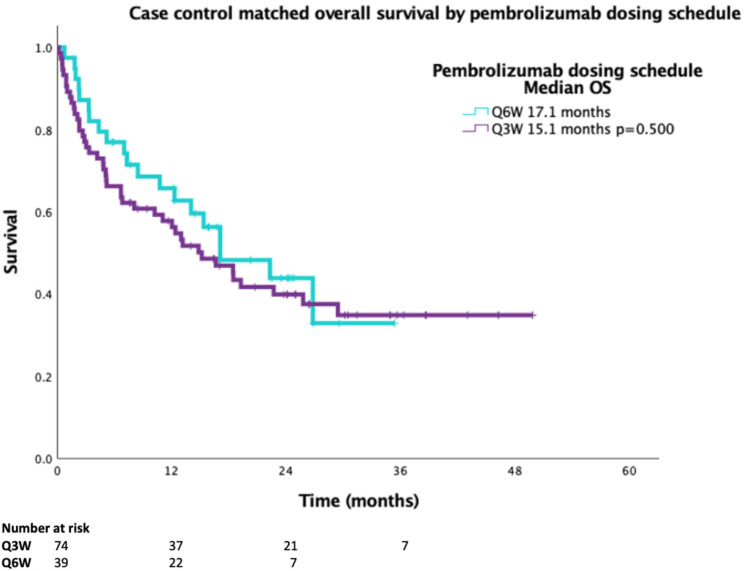
Overall survival of 2:1 case-matched cohort (age ± 5 years, sex, PS and duration on pembrolizumab ± 2 months) for Q3W and Q6W schedule (*n* = 113).

A 2:1 case-matched analysis was performed, controlling for sex, age ± years, PS and duration on pembrolizumab ± 2 months for Q3W vs.Q6W for OS (*n* = 113). Baseline characteristics of the case-matched cohort demonstrated no statistical difference between smoking status or histology (Table 3). The case-matched median OS for Q3W dosing was 15.1 months compared to 17.1 months for Q6W dosing, with a HR 0.834 (95%CI 0.490–1.417, *p* = 0.500) (Figure 2).

## 4. Discussion

In our population-based study, there was no OS difference in patients with stage IV NSCLC and PD-L1 TPS ≥ 50% who were treated with first-line pembrolizumab and dosed at Q3W or Q6W intervals. This study supports the pharmacokinetic modeling suggesting that pembrolizumab dosing is effective at a Q6W dosing interval in real-world populations. These results are further supported by a case-matched analysis using key prognostic variables for patients diagnosed with advanced NSCLC.

There have been retrospective studies assessing the real-world effects of extended dosing intervals of pembrolizumab in patients with NSCLC and PD-L1 ≥5 0%. The focus of these studies was to examine the safety and toxicity of a higher dose of pembrolizumab over a longer interval, and they have generally noted similar adverse event profiles [15,16,17,18]. Patient outcomes with respect to OS were evaluated in the Netherlands cohort; in the 54 patients who received single-agent immunotherapy, there was no statistically significant difference between single-agent pembrolizumab 200 mg given Q3W vs.400 mg Q6W, congruent with our results. Our study comprises the largest cohort to date that examines the different schedules for delivery of therapy.

Consistent with the results of the KEYNOTE-024 study, a fixed dosing of pembrolizumab 200 mg Q3W is an accepted standard. Pharmacokinetic studies noted that exposures expected for pembrolizumab 400 mg Q6W were similar to the 200 mg and 2 mg/kg Q3W, with similar expected target saturation [9]. The evaluation of exposure–response relationships with pembrolizumab suggest that 2 mg/kg Q3W and 4 mg/kg Q6W result in target engagement of 95% based on the trough concentration, similar to the flat dosing schedules [19]. In Canada, weight-based dosing has prevailed, as this strategy may enable cost savings at respective treatment sites. BC Cancer implemented weight-based dosing initially at Q3W (2 mg/kg, capped at 200 mg) in February 2018, which was then expanded to include Q6W (4 mg/kg, capped at 400 mg) in April 2020.

The limitations of this study include the retrospective design, no available information on other potential biomarkers like STK11 and KEAP1, lack of information on use of other medications like steroids, small number of patients treated on the Q6W dosing interval and short follow-up due to the recent approval of Q6W pembrolizumab dosing in April 2021. There is also a potential selection bias for patients receiving Q6W dosing reflecting favorable disease biology, prompting the selection of an extended dosing interval by the treating oncologist. Additionally, the COVID-19 pandemic began during the time frame for data collection, which may have impacted the chosen dosing interval. We attempted to control for this through the matched cohort analysis, controlling for duration of therapy. The strengths of this study include assessment of a real-world patient population with patient representation from the entire population of BC.

In this retrospective study of 718 patients with advanced NSCLC and PDL1 TPS ≥ 50% treated with first-line pembrolizumab, there was no OS difference when the pembrolizumab dosing schedule was Q3W compared to Q6W demonstrated in multivariate analysis, including age, sex and PS. A 2:1 case-matched analysis that also controlled for these variables and duration of treatment confirmed these findings. The results of our study support the use of Q6W pembrolizumab dosing, which allows for less frequent interactions with the medical system, providing additional choice in regimen for patients, saving time for patients and lessening financial toxicity.

## Figures and Tables

**Table 1:** Baseline characteristics of advanced NSCLC PDL1 TPS ≥50% patients by pembrolizumab treatment schedule.

Entire Cohort (*n* = 718)	Q3W *n* = 677	Q6W *n* = 41	*p*-Value
Age at diagnosis (median) years	70	70	0.957
Sex			0.072
Female	372 (55%)	15 (37%)	
Male	305 (45%)	26 (63%)	
Smoking status			0.274
Never	43 (6%)	1 (2%)	
Former	492 (73%)	29 (71%)	
Current	124 (18%)	8 (20%)	
Unknown	18 (3%)	3 (7%)	
Smoking (median) years			0.596
ECOG PS			0.037
0–1	399 (59%)	23 (56%)	
≥2	260 (38%)	14 (34%)	
Unknown	18 (3%)	4 (10%)	
Histology			0.608
Squamous	138 (20%)	7 (17%)	
Non-squamous	539 (80%)	34 (83%)	

**Table 2:** Univariate and multivariate analysis for OS in advanced NSCLC PDL1 TPS ≥ 50% patients treated with first-line pembrolizumab.

Variable	HR (95% CI) Univariate Analysis	*p*-Value	HR (95% CI) Multivariate Analysis	*p*-Value
Age	1.000 (0.990–1.010)	0.986		
Sex				
Female vs. Male	1.100 (0.917–1.321)	0.304		
ECOG PS				
0–1 vs. ≥2	2.051 (1.707–2.464)	<0.001	2.041 (1.699–2.453)	<0.001
Pembrolizumab Schedule				
Q3W vs. Q6W	0.718 (0.458–1.124)	0.147	0.759 (0.485–1.190)	0.230

**Table 3:** Baseline characteristics of 2:1 case-matched cohort (sex, age ± 5 years, PS and duration on pembrolizumab ± 2 months) for Q3W and Q6W schedule.

Case-Matched Cohort (*n* = 113)	Q3W *n* = 39	Q6W *n* = 74	*p*-Value
Age at diagnosis (median) years	70	70	0.964
Sex			1.000
Female	15 (38.5%)	29 (39.2%)	
Male	24 (61.5%)	45 (60.8%)	
Smoking status			0.116
Never	1 (2.6%)	2 (2.7%)	
Former	28 (71.8%)	59 (79.7%)	
Current	7 (17.9%)	13 (17.6%)	
Unknown	3 (7.7%)	0 (0%)	
Smoking (median) years	40	40	0.688
ECOG PS			1.000
0–1	26 (66.7%)	49 (66.2%)	
≥2	13 (33.3%)	25 (33.8%)	
Histology			0.807
Squamous	7 (17.9%)	16 (21.6%)	
Non-squamous	32 (82.1%)	58 (78.4%)	

## Data Availability

Data will be made available upon reasonable request and ethics review.
